# In vivo surface-enhanced Raman scattering techniques: nanoprobes, instrumentation, and applications

**DOI:** 10.1038/s41377-024-01718-5

**Published:** 2025-02-11

**Authors:** Hyejin Chang, Won Hur, Homan Kang, Bong-Hyun Jun

**Affiliations:** 1 Division of Science Education, Kangwon National University, Chuncheon, 24341 South Korea; 2 Gordon Center for Medical Imaging, Department of Radiology, Massachusetts General Hospital and Harvard Medical School, Boston, MA 02114 USA; 3 Department of Bioscience and Biotechnology, Konkuk University, Seoul, 05029 South Korea

**Keywords:** Optical sensors, Imaging and sensing, Nanoparticles, Nanophotonics and plasmonics

## Abstract

Surface-enhanced Raman scattering (SERS) has emerged as a powerful tool in various biomedical applications, including in vivo imaging, diagnostics, and therapy, largely due to the development of near-infrared (NIR) active SERS substrates. This review provides a comprehensive overview of SERS-based applications in vivo, focusing on key aspects such as the design considerations for SERS nanoprobes and advancements in instrumentation. Topics covered include the development of NIR SERS substrates, Raman label compounds (RLCs), protective coatings, and the conjugation of bioligands for targeted imaging and therapy. The review also discusses microscope-based configurations such as scanning, widefield imaging, and fiber-optic setups. Recent advances in using SERS nanoprobes for in vivo sensing, diagnostics, biomolecule screening, multiplex imaging, intraoperative guidance, and multifunctional cancer therapy are highlighted. The review concludes by addressing challenges in the clinical translation of SERS nanoprobes and outlines future directions, emphasizing opportunities for advancing biomedical research and clinical applications.

## Abstract (web summary)

Near-infrared surface-enhanced Raman spectroscopy enables advanced in vivo imaging, diagnosis, and multifunctional biomedical applications.

## Graphical abstract

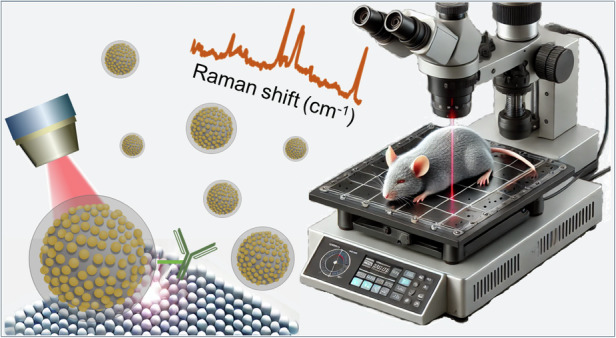

## Introduction

Surface-enhanced Raman scattering (SERS) is a well-known phenomenon in which the Raman signal produced by the inelastic scattering of molecules is significantly amplified near rough metallic surfaces or nanostructures. SERS spectroscopy has emerged as a powerful analytical and sensing technique for diagnostics, drug discovery, and nanomedicine^1–6^. The broad acceptance of this method stems from its informative and non-invasive nature, along with simple yet highly precise measurements.

**Fig. 1:**
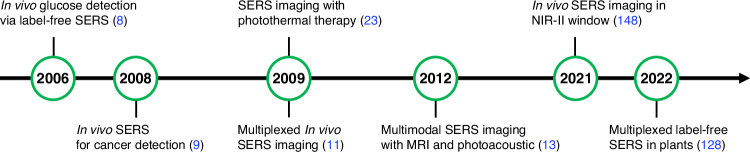
The chronological progression of SERS-based in vivo applications. Corresponding citations are in parentheses

Current clinical imaging methodologies include ultrasound (US), X-ray computed tomography (CT), single photon emission computed tomography (SPECT), magnetic resonance imaging (MRI), and positron emission tomography (PET)^7^. Each of these techniques offers versatility across various medical applications; however, it has certain limitations. For example, X-ray CT, SPECT, and PET rely on ionizing radiation, posing potential health risks from repeated exposure. MRI and PET incur substantial costs and have inherent limitations in spatial resolution. US and X-ray CT are more commonly used due to cost-effectiveness; however, they often only probe basic anatomy and lack advanced multiplex capabilities^7^. Given these limitations, there has been a significant interest in exploring alternative imaging techniques. During the late 2000s, significant attention was drawn to SERS in vivo applications, presenting new avenues for biomedical imaging and molecular detection (Fig. 1). In 2006, in vivo glucose sensing was reported as the first SERS application^8^. Two years later, the Nie group reported non-invasive cancer detection using epidermal growth factor (EGF)-targeted gold nanoparticles (Au NPs) in human head and neck squamous cell carcinoma-bearing nude mice^9^. Concurrently, the Gambhir group demonstrated multiplex detection using Au NPs labeled with small molecules containing benzene rings^10^. These pioneering studies have paved the way for various approaches to enhance SERS nanoprobes’ sensitivity, biocompatibility, and multifunctionality for in vivo applications^6,11–22^. Various noble metal NPs are promising candidates for SERS nanoprobes if they have the desired size, potential targetability, and multimodality through suitable modifications. The development of localized surface plasmon resonance (LSPR)-tuned plasmonic NPs with anisotropic or branched morphologies, such as nanorods^12,23^, nanostars^18,24,25^, and nanoshells^15,16^, has enriched the utilization of near-infrared (NIR) windows for advanced sensitivity and resolution.

**Fig. 2:**
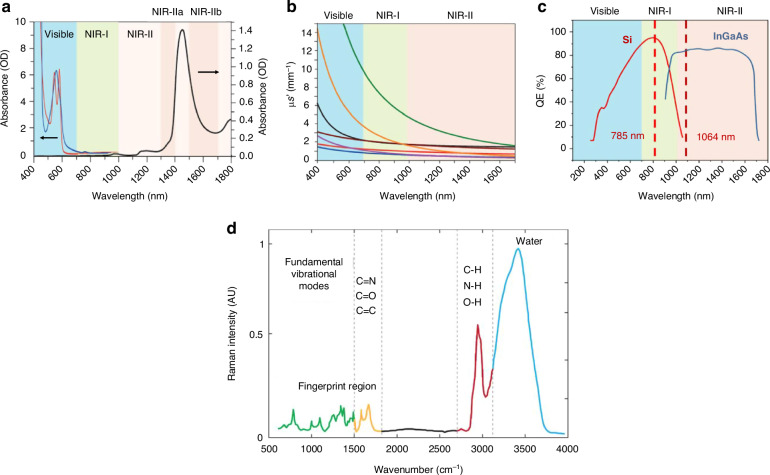
Motivation for NIR SERS-based in vivo application. **a** Absorbance of oxygenated (red curve) and deoxygenated (blue curve) hemoglobin in the visible and NIR spectrum, together with water absorbance (black curve) at 1400–1500 nm. **b** Scattering coefficients as a function of wavelength in the 400–1700 nm region, for various biological tissues and intralipid scattering tissue phantom. Reproduced from ref. ^26^ with permission from Springer Nature. **c** Sensitivity curves (Quantum efficiency as a function of wavelength) for typical cameras based on silicon (Si) or indium gallium arsenide (InGaAs), which are sensitive in the first and second windows. The excitation wavelength at 785 nm and 1064 nm are indicated by vertical dotted lines. Reproduced from ref. ^28^ with permission from Elsevier. **d** Raman spectrum of P22 virus in H_2_O buffer. The spectrum was obtained with 514.5 nm laser excitation. The main structurally informative Raman bands occur in the 600–1800 cm^−1^ interval. Reproduced from ref. ^58^ with permission from John Wiley and Sons

Using visible light (<650 nm) for in vivo imaging generally results in low sensitivity and poor signal reproducibility. This limitation arises because the abundant chromophores in animal tissues lead to strong absorption, autofluorescence, and heavy scattering, as shown in Fig. 2a, b, resulting in a low penetration depth and high background noise levels for in vivo imaging^26–28^. In contrast, the NIR window is more suitable for in vivo applications because the endogenous tissue absorption coefficients in the NIR region are two orders of magnitude lower than those for ultraviolet and visible light. There are two windows, the first NIR (NIR-I, 650–900 nm) and the second NIR (NIR-II, 1000–1700 nm), for in vivo imaging because of nanomaterial and instrumental developments^29^. Initially, cost-effective and widely available instrumentation for the NIR window required for in vivo SERS studies involved Raman systems using 785 nm diode lasers and silicon (Si) detectors. Thus, the starting point for in vivo SERS imaging was the NIR-I region. With advances in low-loss spectrographs and indium gallium arsenide (InGaAs) charge-coupled devices (CCDs), in vivo imaging research, including fluorophores and SERS nanoprobes, has shifted to operating in the NIR-II window. The detection efficiency for silicon detectors diminishes beyond 900 nm, while for InGaAs detectors, it sharply increases after 1000 nm (see Fig. 2c). Consequently, there is a restricted detectable range between 900 and 1000 nm.

Building on these foundational insights, we explore the current state-of-the-art in vivo SERS techniques applied in cancer diagnostics and therapeutic interventions. We examine the unique properties of SERS nanoprobes and their specific applications in in vivo detection and imaging. Furthermore, we detail various instrumentation aspects that are crucial for the practical implementation of in vivo detection, providing a comprehensive overview of the technological and methodological advancements in this field.

This review distinguishes itself from previous reviews by offering a more comprehensive overview of SERS-based in vivo applications. Unlike previous reviews that focused primarily on specific areas^7,30,31^ such as SERS probe design and cancer imaging, this review addresses a broader scope including the components of NIR SERS probes, advancements in Raman instrumentation, and applications in disease diagnostics and therapy. Additionally, it emphasizes clinical translation through discussions on innovative imaging techniques (e.g., widefield and fiber-optic configurations) and explores multiplex imaging and intraoperative guidance. This broader coverage offers a more holistic perspective on SERS technology’s current state and future directions, particularly for biomedical research and clinical applications.

## In vivo SERS detection strategies

SERS-based in vivo detection and imaging can be classified into two types: (1) direct detection, also known as the label-free SERS approach, and (2) indirect detection using SERS labels (referred to as *SERS nanoprobes* in this review) comprising nanosubstrates, Raman label compounds (RLCs), protective shells, and targeting ligands^32,33^. In this section, we discuss these two strategies regarding their inherent advantages and limitations, focusing on describing SERS nanoprobes as a key analytical and imaging tool for in vivo SERS applications.

### Direct detection strategy: label-free SERS

Direct SERS detection and imaging utilize the intrinsic vibrational signatures of molecules to provide detailed chemical and structural information, enabling the identification and quantification of biomolecules such as proteins^3,34^, nucleic acids^1,2,35,36^, and pathogens^37,38^ without the use of additional Raman labeling and probes. Direct SERS detection is advantageous owing to its inherent simplicity, which enables expedited analytical procedures. The streamlined property of the label-free detection process allows accelerated analyses, which are particularly beneficial in clinical settings where time constraints prevail. Furthermore, it minimizes potential side effects or toxicity concerns associated with external labeling agents.

Direct detection using SERS faces several limitations, primarily the weak Raman signals caused by the relatively low Raman scattering cross-section and surface affinity of biomolecules. Additionally, the relatively low concentrations of target molecules within complex biological matrices result in weak Raman signals with significant background noise, complicating the detection and identification of specific molecular signatures. In the spectra of macromolecules with similar repeating units, such as proteins or DNA, most of the main peaks overlap, necessitating extensive data processing for quantitative or qualitative analysis. Furthermore, a high degree of freedom in target movement can interfere with the rapid identification of target molecules in biological matrices, resulting in low reproducibility. Despite these sensitivity and background interference challenges, direct SERS detection remains a significant area of research and development. Ongoing studies aim to optimize SERS-active substrates, advance data processing algorithms, and improve overall sensitivity and specificity. As a result, direct SERS detection is becoming increasingly prominent in biomedical research and clinical diagnostics, serving as a comprehensive toolkit for SERS-based in vivo applications.

### Indirect detection strategy: targeted SERS nanoprobes

Indirect detection using SERS nanoprobes has been developed to overcome the challenges associated with direct SERS detection and enhance the applicability of SERS in biological imaging and quantitative sensing. These nanoprobes typically utilize the signals encoded with RLCs on metallic NPs to indicate binding events between specific targets and ligands attached to the nanoprobes. As promising imaging agents, SERS nanoprobes generate optical signals by binding to biomarkers in tumors or tissues, providing specific biological information and aiding in the complete removal of tumors. The high heterogeneity of biomarker expression, which can vary significantly among cancer subtypes, patients, and even within a single tumor, presents a substantial challenge for cancer imaging. SERS nanoprobes can potentially enhance the sensitivity and specificity of in vivo diagnostics and imaging due to their ability to detect multiple biomarkers simultaneously. This multiplexing capability allows for more precise and comprehensive detection of cancer biomarkers, improving diagnostic accuracy and treatment outcomes.

The development of quantitative and sensitive SERS nanoprobes has been of great interest in the fields due to the several advantages compared to other analytical techniques such as fluorescence tags^32,39,40^: (1) a large multiplex detection capacity coming from a narrow Raman band width (<2 nm) under single wavelength photoexcitation, (2) stable signals without photobleaching, (3) relatively easy surface modifications, and (4) low auto-fluorescence background in NIR excitation, and (5) high sensitivity due to optical tuning of plasmonic NPs to the NIR window. These unique characteristics have led to the successful use of SERS nanoprobes for in vivo optical imaging.

**Fig. 3:**
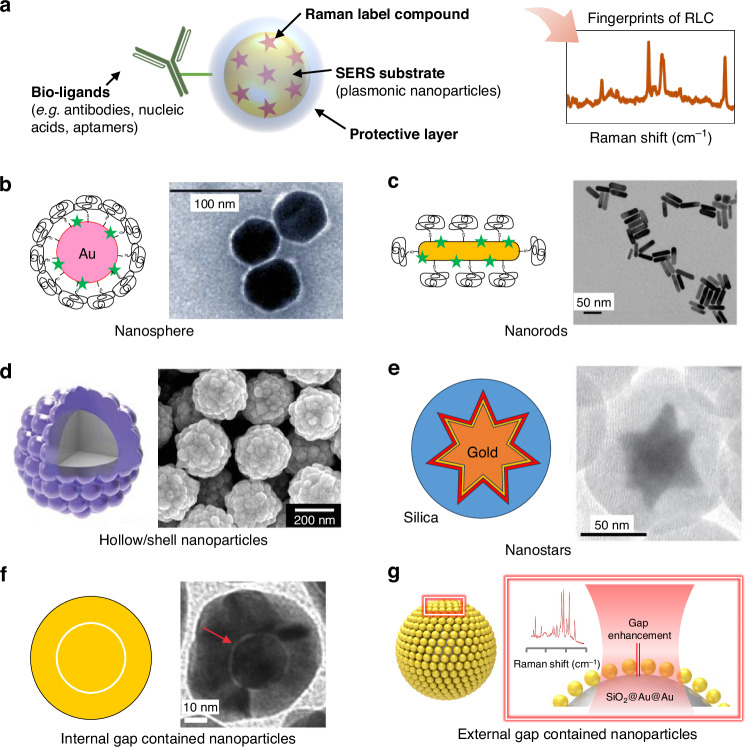
SERS nanoprobes for in vivo application. **a** Schematic illustration of a typical SERS nanoprobe composed of plasmonic nanoparticles as SERS substrate, Raman label compounds, protective layer and bioligands. Various types of plasmonic nanoparticles utilized for in vivo SERS nanoprobes. **b** Nanospheres. Adapted from ref. ^9^ with permission from Springer Nature. **c** Nanorods. Adapted from ref. ^45^ with permission from Copyright 2020 American Chemical Society. **d** Hollow/shell nanoparticles. Adapted from ref. ^48^ with permission from Royal Society of Chemistry. **e** Nanostars. Adapted from ref. ^18^ with permission from the American Association for the Advancement of Science. **f** Internal gap contained nanoparticles. Adapted from ref. ^21^ under (CC BY 4.0). **g** external gap contained nanoparticles. Adapted from ref. ^22^ under (CC BY 4.0)

Typically, SERS nanoprobes are designed to comprise four components as shown in Fig. 3a: (1) SERS substrates, (2) extrinsic label molecules referred to as RLCs attached on the surface of SERS substrates, (3) protecting layers for physical and chemical stability of inner structures such as poly(ethylene glycol) (PEG) and silica, and (4) bio-ligands for targeting a target molecule, such as antibody, aptamer, and DNA^39,40^. Several factors must be considered to develop SERS nanoprobes for successful in vivo imaging. First, a suitable plasmonic NP is required to generate a localized electric field under appropriate laser conditions (wavelength and power) to provide a strong SERS signal and high sensitivity. Second, the spectral simplicity of the nanoprobes derived from RLCs must be designed to enhance the multiplexing capacity. Third, evaluating the toxicity of these nanoprobes is crucial for potential clinical applications. Fourth, the stability of nanoprobes under physiological conditions and their specific binding to target molecules such as antibodies, aptamers, and DNAs are essential factors to consider.

In this section, we focus on SERS nanoprobes’ basic elements and features, particularly in vivo sensing and imaging.

#### SERS nanoprobes: SERS substrates

SERS nanoprobes are engineered to amplify the Raman signals of labeled molecules adsorbed onto SERS substrates, enabling highly sensitive detection and characterization. Various SERS substrates are available, each with unique characteristics and fabrication methods tailored to their specific sizes, shapes, and surface properties. These substrates are optimized to enhance their plasmonic properties and maximize the SERS effects.

The dominant contribution to the overall SERS effect is electromagnetic (EM) enhancement^40^. When incident light interacts with LSPR in nanoscale noble metal surfaces, the local strength of EM field increases dramatically relative to the incident light intensity. The optical excitation of LSPR usually occurs in the visible to NIR region in the case of Ag, Au, and Cu NPs. Although this EM field enhancement is known to be a long-range effect and highly heterogeneous, depending on the distance from the surface and local structures, such as sharp edges or tips, the ensemble-averaged enhancement of commonly used NP-type substrates^41^ is reported to be 10^6^. Designing plasmonic NPs and adjusting their size, shape, and plasmonic coupling to align the LSPR peak in the NIR region are critical for the sensitivity and in vivo applications of SERS nanoprobes.

Traditionally, spherical Au and Ag NPs have been employed as SERS substrates due to their LSPR absorption occurring in the visible region, which aligns well with readily available lasers. These NPs benefit from well-established synthetic methods and exhibit remarkable stability. For biomedical applications in the NIR region, Au NPs are preferred over Ag NPs due to their superior biological stability, biosafety, and optical properties.

The initial application of in vivo SERS imaging utilized Au NP-based nanoprobes with diameters of ~60 nm (Fig. 3b)^9,10^. The UV-Vis extinction spectrum revealed that the LSPRs of simple and (quasi-)spherical Au NPs did not show effective interactions with photons of 785 nm wavelength, which is commonly used for in vivo imaging. Nevertheless, larger Au NPs with enhanced scattering efficiency and increased surface area, combined with using resonant dyes as RLCs, can improve signal intensity, enabling successful in vivo imaging.

To develop sensitive in vivo SERS nanoprobes, various advanced nanostructures with strong LSPR in the NIR region have been reported^6,15,16,18,20,22,23,42–44^. Au nanorods (NRs) have garnered significant attention owing to their tunable plasmon resonance, which depends on their aspect ratio. The Au NRs exhibited two absorption bands attributed to different dipole LSPR modes: transverse and longitudinal. By increasing the aspect ratio of the Au NRs, the longitudinal band could be shifted towards the NIR window. Therefore, Au NRs can serve as effective NIR absorbers and have been successfully applied for in vivo imaging (Fig. 3c)^12,23,45^. An initial report on using Au NRs for in vivo SERS applications involved the simultaneous utilization of SERS imaging and photothermal therapy (PTT) of tumors under NIR irradiation^23^.

Nanoshells, structured with a hollow interior, have been developed to shift plasmonic band features to the NIR region, showing effectiveness for in vivo imaging^20,46–48^. Hollow Au nanoshells can be prepared by depositing Au with concomitant Ag or Co template oxidation. The LSPR of the nanoshells can be adjusted by changing the size of the template and the shell thickness. When nanoshells are prepared with an encapsulated dielectric core, usually silica, the structures can feature a plasmon band shifted to the NIR region by altering the core diameter and shell thickness^49,50^. To avoid the time-consuming multistep processes of Au nanoshells, a one-step synthetic route to uniform bumpy Ag nanoshells has been reported (Fig. 3d)^48^. This seedless synthesis, using a dielectric silica core, produced thickness-controlled bumpy nanoshells that exhibited NIR plasmonic absorption. Although general concerns remain regarding the biotoxicity of silver compared to gold, hematologic evaluation results demonstrated that the testing dose (<50 mg kg^−1^) of the PEGylated Ag nanoshell probes did not cause any in vivo toxicity.

Nanostars have gained attention for in vivo SERS sensing because they typically generate strong SERS signals at their sharp edges^18,24,25,51,52^. It is well known that EM fields and the excitation cross section dramatically increase on the sharp edges or tips of plasmonic NPs, where the so-called “hot spot”^51^. The energies of the tip plasmons are closely correlated with the aspect ratio of the tip and exhibit a red shift with increasing tip length. Thus, by precisely tuning the tip structures, significant signal enhancement can be achieved. This enhancement results from the combined effects of the concentrated local field and the red-shifted plasmon mode, which matches NIR excitation. SERS nanoprobes fabricated with a 75-nm star-shaped gold core that demonstrated LSPR in the NIR window and a resonant RLC (IR-780 perchlorate) showed a detection limit ~400-fold lower than that of spherical and non-resonant Au NPs (Fig. 3e)^18^.

The development of reproducible and homogeneous SERS nanoprobes capable of creating controlled hotspots is a critical challenge^16,53,54^. These hotspots, characterized by highly concentrated EM fields, manifest predominantly at the tips, edges, and within nanogaps. These hot spots are known to contribute to EM enhancement of up to ${10}^{11}$, allowing single-molecule sensitivity. Internal nanogap-containing NPs are promising for biomedical applications, benefiting from highly uniform and strong signal enhancement, and effective prevention of interference effects in the surrounding media (Fig. 3f)^21,53,55,56^.

The Ye group developed gap-enhanced Raman tags (GERTs) with a smooth external shell designed with built-in RLCs for off-resonance NIR laser excitation^56^. GRETs showed high photostability attributed to reduced photothermal effects across diverse conditions, encompassing saline, fetal bovine serum, and varying pH environments. Subsequently, GERTs with petal-like shell structures were developed to increase the immobilization surface area and Raman cross section^21^. Benefiting from the high brightness of GRETs, high-contrast wide-area imaging (3.2 × 2.8 cm²) of the hind-limb sentinel lymph nodes was achieved within 52 s. This demonstrates the potential for rapid widefield imaging made possible by advancements in highly sensitive SERS nanoprobe technology.

External gap structures created through the dimerization or assembly of NPs represent an effective strategy for generating hotspots. When two plasmonic NPs are positioned nearby, a highly concentrated electric-field enhancement occurs between the NPs. However, this enhancement is highly sensitive to the gap distance^57^, making it crucial to fabricate SERS substrates with effectively coupled and well-controlled nanostructures for high sensitivity and uniformity. In this regard, three-dimensional (3D) core-satellite assembled structures^16,22,44,54^, such as Ag Shell−Au satellite hetero-nanostructure^16^, offer uniform signal enhancement and independence from laser polarizations due to the isotropic SERS activity.

Recently, Au‑assembled silica NPs (SiO_2_@Au@Au NPs) were reported for in vivo SERS applications (Fig. 3g)^22^. SiO_2_@Au@Au NPs with six different sizes of Au NPs were prepared using the seed‑mediated growth method, and the nanogaps between Au NPs on the silica surface could be controlled from 4.16 to 0.98 nm for effective SERS hot spots. Maximum SERS enhancement factor of SiO_2_@Au@Au NPs reached ~3.8 × 10^6^ under 785‑nm photoexcitation, providing detectable SERS signals of SiO_2_@Au@Au_500_ nanoprobes at a concentration of 16 μg/mL in animal tissue specimen at a depth of 7 mm. Another NIR-SERS nanoprobe involving Au/Ag alloyed hollow-shell assemblies on silica cores was reported^15^, providing tunable plasmon bands from 480 to 825 nm by varying the diameter of the hollow shell interiors and the Au:Ag atomic ratio.

#### SERS nanoprobes: Raman label compounds (RLC)

Efficient RLCs are crucial for fabricating in vivo SERS nanoprobes because they significantly affect the sensitivity and multiplexing capacity. NIR excitation, although preferred in in vivo applications to reduce noise and background signals, cannot produce strong enough signals because scattering efficiency decreases with longer wavelengths following the *λ*^−4^ relationship. Hence, RLCs with inherently large Raman cross-sections are essential for developing sensitive SERS nanoprobes.

Molecules with a π-electron system and high polarizability exhibit large Raman cross-sections, and their signal can be further enhanced 10–100 fold through surface-enhanced resonance Raman scattering (SERRS) when laser line matches the electronic transition of the molecule. In early in vivo studies, SERS nanoprobes typically used UV-Vis active chromophores (e.g., rhodamine-6G, malachite green isothiocyanate, and crystal violet), limited to in vivo imaging applications. The cyanine derivative 3,3’-diethylthiatricarbocyanine (DTTC), a NIR dye, was considered better for in vivo applications^9^, but their positive charge can reduce surface affinity, thereby potentially lowering the sensitivity of SERS nanoprobes. Effective RLCs also require functional groups such as thiols, isothiocyanates, or amines that facilitate surface attachment, enabling high sensitivity, long-term stability, and further modification of SERS nanoprobes.

One significant advantage of SERS nanoprobes over fluorescent dyes and quantum dots is their superior multiplexing capacity for in vivo imaging. To harness this potential, RLCs in libraries should possess distinct spectral bands that can be easily resolved. Most organic chromophores with large Raman cross-sections exhibit prominent Raman bands in the 600–1800 cm^−^¹ fingerprint region (Fig. 2d)^58^. This area exhibits a high population density of multiple bands, posing challenges in distinguishing individual signals. Enhancing multiplex applications would benefit from investigating NIR resonant dyes featuring unique structures and clearly separated bands. This approach would enhance the clarity and specificity of multiplexed SERS imaging, enabling more precise and comprehensive analyses.

Some research groups have made advances in RLC libraries to improve their sensitivity, chemical and physical stability, and multiplex capacity of RLC libraries instead of using commercially available compounds^17,59–64^. The Chang group reported a lipoic acid-containing NIR active tricarbocyanine library with NIR absorption properties and good chemical stability in aqueous media^59^. The tricarbocyanine core was used as an accessible NIR structure, and the dithiols of lipoic acid linker functioned as anchoring groups to the Au NP surface. The screened compound (CyNAMLA-381) showed a 12-fold higher sensitivity than DTTC. The same group reported the continuous development of RLC libraries for constructing highly sensitive and stable NIR SERS nanoprobes, demonstrating multiplex-targeted in vivo tumor detection^60,65^. The Kircher group also developed thiophene-substituted chalcogenopyrylium dyes^17^, benefiting from strong resonant SERS signals in the NIR region and multiple selenium or sulfur groups with a strong affinity to gold surfaces.

High background signals from biological molecules such as peptides, proteins, and cytochrome C can complicate the interpretation of Raman spectra. Thus, bioorthogonal RLCs with vibrational modes in the Raman-silent region (1750–2750 cm^−1^, Fig. 2d) are gaining interest^45,66–69^. The Tang group constructed a bioorthogonal SERS nanoprobe labeled with four different cysteine analogs^62,69^. Raman peaks corresponding to alkyne, azide, and cyano moieties were detected at 2010, 2142, and 2209 cm^−1^, respectively^6,70^. These signals were successfully applied in the SERS nanoprobe development, leading to specific tumor recognition in living mice. The same group further reported the application of bioorthogonal SERS nanoprobes for in vivo imaging combined with PTT^45^. Expanding the RLC library with improved stability and sensitivity is considered one of the keys to better multiplex imaging^22,63,64^, which will be discussed in “Biomolecule Screening and Multiplex Imaging”.

#### SERS nanoprobes: protecting layers and conjugation of bioligands

Protective layers and bioconjugation of SERS nanoprobes are prerequisites for biocompatibility, stability, target specificity, and clearance, leading to successful in vivo applications. The chemical and physical stability of SERS nanoprobes directly affects signal sensitivity and reliability, especially in complex biological media with high ionic strength and dynamic changes, which can cause damage or severe agglomeration of SERS nanoprobes.

An effective protective layer preserves the core structure of SERS nanoprobes, prevents the detachment or chemical transformation of RLCs, and enhances biocompatibility. It can also facilitate targeted molecule conjugation and influence cell membrane interactions, ultimately affecting the binding specificity^71^. Common protective agents include polymers^9,12,23,48,52,70,72–75^, silica^6,10,15,17,21,56,76,77^, proteins^20,78^, and lipid bilayers^79,80^, and the most widely used methods involve PEG or silica layers. PEG can provide hydrophilicity, flexibility, and electrical neutrality. PEGylation, coating NPs with PEG, prevents protein adherence and macrophage removal, leading to a prolonged circulation time in vivo^9,81–83^. Further surface modifications, including the conjugation of targeting bioligands, can be achieved using thiolated-PEG molecules with a chemically active distal terminal group (e.g., carboxyl or amine).

The silica encapsulation of RLC-adsorbed NP cores provides strong stability in biological environments^84,85^. The usual method for silica coating is based on the classic Stöber method using tetraethyl orthosilicate to form a mesoporous silica layer. The hydroxyl groups on the silica surface facilitate further functionalization, enabling conjugation of diverse bioligands and heterobifunctional silane linkers (e.g., 3-aminopropyltrimethoxysilane; APTMS) to introduce additional surface modifications such as PEGylation. However, surface modification processes can compete for metal-binding sites, limiting RLC signal intensities and polymer grafting densities. To address this issue, bifunctional molecules that act as both RLC and silica precursors have been suggested for silica encapsulation in nanoshells^86^. In this approach, aminoalkyl alkoxy silanes such as APTMS serve as silica precursors and are covalently bonded with derivatives of mercaptobenzoic acid, which act as RLCs.

Multifunctional polymers also serve as biocompatible coatings and NIR-active reporters^70,73^. A dye-modified hydrophilic polymer was developed by modifying commercially available NIR dyes with 4-aminothiophenol through the modulation of the polymeric precursor, poly(pentafluorophenyl methacrylate) (pPFMA)^73^. Conducting polymers such as polyaniline (PANI) and polypyrrole (PPy), which can act as both surface coating reagents and NIR active RLCs, have also been reported as alternatives to the uncontrolled silica encapsulation process^70^. These polymeric dyes commonly showed remarkable improvements in chromophore loading efficiency, structural stability, and biocompatibility. A facile silica coating method for NIR dye-labeled SERRS nanoprobes was described, employing waxberry-like Au NPs enclosed within lipid bilayers^87^. This approach led to the achievement of outstanding stability in the nanoprobes.

Effective bioconjugation methods are crucial in attaching targeting ligands to SERS nanoprobes, facilitating sensitive detection and precise localization of target molecules. It is essential to carefully select conjugation chemistry and materials to ensure stability and prevent potential toxicity. Biocompatible options such as thiol-terminated PEG and amine-functionalized silica are advantageous, as they remain stable under physiological conditions. Targeting ligands should exhibit high affinity and specificity to minimize off-target effects, enhancing the precision of biomolecule detection and localization. By leveraging these considerations, researchers can optimize the performance and safety of SERS nanoprobes for diverse biomedical applications.

Various bioconjugation methods include thiol, carbodiimide, maleimide, and click chemistry. Thiol-functionalized targeting ligands bind to gold surfaces via gold-thiol chemistry^88^. Carbodiimide reagents, such as N-ethyl-N′-(3-dimethylaminopropyl)carbodiimide (EDC), activate carboxyl groups for amide bond formation with primary amines on NP surfaces^6,89^. Maleimide chemistry couples maleimide-functionalized ligands to thiol-functionalized NPs^90^, while click chemistry couples azide- and alkyne-functionalized ligands via azide-alkyne cycloaddition^91–93^. The choice of method hinges on the targeting ligand and nanoprobe surface chemistry, which require careful optimization for effective biomarker detection.

In summary, effectively utilizing SERS nanoprobes across different applications necessitates meticulous attention to surface modifications, including the protective layers coatings and ligand conjugations. Key considerations include: (1) ensuring that the fabrication process is straightforward and manageable, (2) enabling facile modulation of nanoprobe functionality through surface functionalization and treatment, (3) maintaining high sensitivity and excellent resolution for easy particle identification, and (4) ensuring stable preservation of the shape and labeling of synthesized and fabricated particles. These considerations are crucial to enhance the versatility and reliability of SERS nanoprobes for diverse applications.

## Instrumentation for in vivo SERS application

**Fig. 4:**
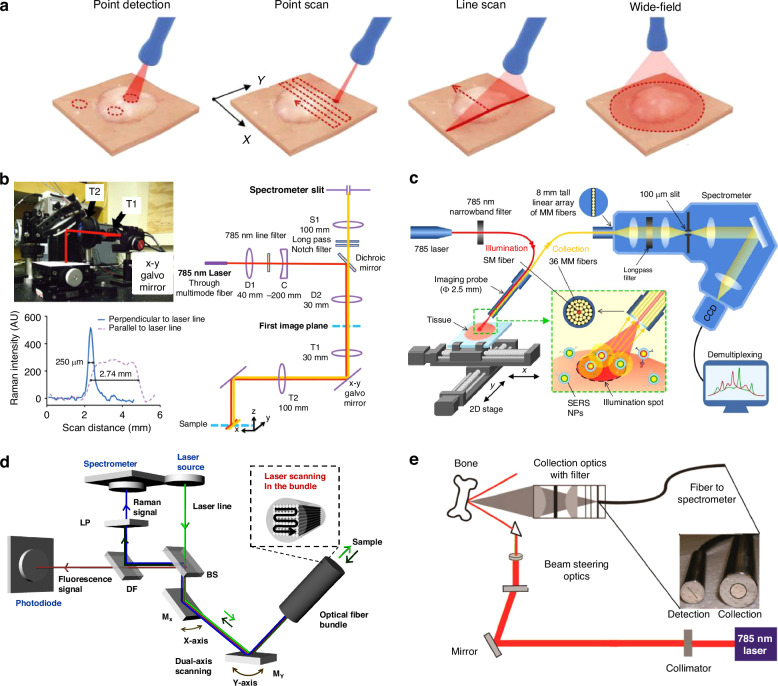
Raman instrumentation for in vivo applications. **a** Schematics of Raman imaging approaches and (**b**–**e**) various optical systems for in vivo Raman imaging. Reproduced from ref. ^94^ with permission from IEEE. **b** Optical design of small animal Raman imaging instrumentation (SARI) system for wide-area mapping and characterization and line-spread function. SARI performs a line-scanning in which a laser line is raster scanned along the *x* and *y* axes. Reproduced from ref. ^14^ with permission from National Academy of Sciences of the United States of America. **c** Schematic of Raman-imaging system based on white-light endoscopy. Rotating mirror by a brushless DC motor sweeps the collimated beam by 360 degrees, enabling luminal imaging of the colon wall. Reproduced from ref. ^97^ under (CC BY 4.0). **d** Optical design of fluorescence-Raman endomicroscopic system (FRES) using 532 nm laser. M_x_ and M_y_ = oscillating mirror for *x*-axis and *y*-axis, respectively. BS beam splitter, DF dichroic filter, and LP long pass Rama edge filter. The signal was collected into two divided sections by DF; fluorescence detection region (≥2,000 cm^−1^ above from a Rayleigh line) and Raman detection region (from 1250 cm^−1^ to 2000 cm^−1^). Reproduced from ref. ^19^ under (CC BY 4.0). **e** Schematic of surface-enhanced spatially offset Raman spectroscopy (SESORS) instrumentation with 785 nm laser. The inset photograph shows collection and detection ends of fiber. The nanoprobes with trans−1,2-bis(4-pyridyl)-ethylene (BPE) were injected into the tissue and SESORS spectra were collected. Reproduced from ref. ^112^ with permission from Copyright 2013 American Chemical Society

The effectiveness of in vivo SERS detection and imaging depends not only on the sensitivity and specificity of SERS nanoprobes but also on the optical instruments’ capabilities. The key factors to consider in Raman instrumentation include the laser wavelength and power, objective lens, spectrometer parameters (including the grating and detector), and acquisition time. Different detection methods, such as single-point detection, point scanning (point-by-point mapping), line scanning, and widefield imaging, as depicted in Fig. 4, offer various advantages depending on the application. Therefore, selecting an appropriate imaging system compatible with the SERS nanoprobes is essential for successful in vivo SERS implementation.

Although commercial Raman microscopes provide high-resolution imaging at the cellular or molecular level^42,48,55,76^, their long acquisition times limit their use in submillimeter-sized tissues. Therefore, microscope-based Raman systems with point detection are not ideal for in vivo measurements. A comprehensive evaluation of the imaging approaches and optical setups, including selecting a suitable microscope and filter, is crucial for effective in vivo SERS detection. This section discusses the instrumental setups for in vivo SERS imaging, focusing on scanning-based imaging, widefield imaging, and the endoscopic approach. We also cover the key considerations for designing a Raman system for in vivo applications, such as the excitation source, illumination and collection system, and detector.

### General considerations

Selecting an appropriate excitation wavelength within the biological window is crucial to minimize laser absorption, scattering, and autofluorescence in biological samples. The 785 nm laser is widely favored due to its balanced sensitivity, signal intensity, overall performance, cost-effectiveness, and capability of effectively penetrating tissues. For detection in the NIR-II window, wavelengths such as 1064 nm can be considered. However, caution is necessary to avoid tissue heating. Laser intensity must be meticulously controlled to prevent tissue damage and adhere to the maximum permissible exposure (MPE) limits specified by the American National Standards Institute (ANSI) for safe use in biomedical applications^94,95^. The MPE values depend on laser wavelength, exposure time, and the biological target (eye or skin). For visible wavelengths, the MPE for skin is 0.2 W/cm^2^, which increases exponentially between 700 nm and 1050 nm to a maximum of 1 W/cm^2^ according to ANSI Z136.1. However, as the beam size increases, the permissible irradiance decreases due to cumulative thermal effects caused by the strong tissue absorption at these wavelengths^96^.

The selection of objectives in the illumination and collection paths is critical in determining laser spot size and power density, affecting sample degradation and tissue damage. In a Raman microscope, the laser spot size depends on both the laser wavelength and the numerical aperture (NA) of the objective lens. For instance, with a 785 nm laser and a 0.90/100× objective, the spot size is ~1064 nm. Higher NA objectives result in smaller spot sizes and higher power density. In the context of in vivo applications, it is advisable to use lower magnification objectives (e.g., 10×, 20×) with moderate NA (ranging from 0.25 to 0.50). These objectives offer longer working distances (typically several millimeters), allowing deeper tissue penetration while maintaining adequate spatial resolution. They minimize sample damage, enabling imaging of live tissues without overheating or photodamage. Conversely, higher magnification objectives (e.g., 40×, 60×) with NA values exceeding 0.7 provide enhanced spatial resolution. However, due to their shorter working distances (often less than 1 mm), they are better suited for thin ex vivo tissue sections or surface-level imaging. For in vivo Raman applications, fiber optic probes are particularly advantageous. They allow flexible light delivery and collection in challenging-to-access areas, facilitating targeted and minimally invasive measurements. By coupling fiber probes with lower NA optics, researchers can achieve deeper penetration while preserving signal strength, which is ideal for in vivo studies.

The choice of a CCD detector for in vivo SERS imaging depends on several critical factors, including spatial resolution, signal intensity, and sensitivity. Smaller pixel sizes in CCDs offer better spatial resolution but may reduce sensitivity, necessitating a careful balance. Specifically, for 785-nm excitation, CCD detectors typically exhibit good sensitivity in the 750–1050 nm range. When optimizing performance, factors such as spectral range, noise levels (including dark noise, readout noise, and shot noise), and cooling capabilities should be considered.

Electron-multiplying CCDs (EMCCDs) are particularly beneficial in low-light Raman imaging. They enhance the signal-to-noise ratio by amplifying weak signals, enabling faster acquisition times and clearer results than conventional CCDs. On the other hand, for 1064-nm excitation, InGaAs detectors are preferred due to their efficiency in the NIR range beyond 1100 nm, although they often provide lower spatial resolution. Larger pixels enhance sensitivity but diminish resolution, making it essential to find a balance between pixel size and the level of detail required. In real-time imaging, rapid acquisition is necessary; however, increased speed may compromise the signal-to-noise ratio. Carefully managing these elements is vital.

### Microscope-based configuration: scanning and widefield imaging

In preclinical imaging, a confocal Raman system can scan small animals at the mapping stage. Early in vivo SERS imaging studies utilized Raman microscopy systems that scanned a laser spot across the samples and recorded the spectra at each (*x*, *y*) point using a linear array CCD detector^10,23^. This setup enabled the subcutaneous imaging of rodent models optimized for detecting SERS probe signals, often using a defocused beam with a low NA objective.

Raster scanning, which employs a laser beam as a point or line, is achieved using a galvanometric scanning mirror or mechanical stage. This technique provides full spectra for each point or line, facilitating the multiplexed detection and quantification of SERS nanoprobes. However, scanning-based imaging often suffers from long integration times and reduced imaging speeds owing to mechanical motion and slow processing rates.

Line scanning has been explored as a promising alternative for overcoming the limitations of point scanning, such as extended acquisition times, low spatial resolution, limited field of view, and challenges in animal handling. Bohndiek et al. developed a line-scanning setup combined with a two-dimensional (2D) electron-multiplying charge-coupled device (Fig. 4b) for fast spectroscopic imaging over a wide field of view (>6 cm^2^), called the small animal Raman imaging (SARI) system^14^. This SARI system improves the imaging speed compared to conventional microscopy systems while maintaining selectivity, multiplexing capabilities, and spectral and spatial resolution.

Wang et al. developed Raman-encoded molecular imaging (REMI) technology (Fig. 4c) for the quantitative ex vivo imaging of multiple biomarkers on fresh tissue surfaces^97,98^. Employing a 785-nm laser with a submillimeter-diameter beam spot by 36 multimode fibers, a custom-designed two-axis stage, REMI translated the tissue sample with a travel range of 50 × 50 mm^2^ and adjustable velocities ranging from 1.3 μm/s to 20 mm/s. Moreover, by combining the 2D raster scanning platform with a spatially offset Raman probe, the system enabled the spectral imaging of large tissue areas with variable spatial resolution, ranging from 0.2 to 1 mm. Initial studies demonstrated the simultaneous quantification of two biomarkers in fresh tissues within a 15-min timeframe through topical application and ratiometric imaging^97^. Subsequent studies involving tumor xenografts and human breast tissues expanded their capabilities to include at least four biomarkers^98^.

Widefield Raman imaging, also called global imaging, provides rapid acquisition of 2D images by using a tunable filter or dichroic beam splitter to illuminate a large area and collect photons from all spatial points on a 2D array detector. This approach enhances imaging speed but compromises molecular fingerprinting, thereby reducing selectivity and multiplexing capabilities. Widefield imaging is most effective with thin samples to achieve high-contrast images and typically involves costly focal plane arrays and fast-tunable filters.

In vivo SERS imaging enables rapid, background-free, widefield imaging using narrow bandpass (BP) filters centered on specific SERS peak location^99^. This method requires only three images (one at the SERS peak and each side of it) per SERS line of interest, significantly reducing the imaging time compared to spectral mapping. Mallia et al. employed this approach to develop a SERS widefield imaging system with 785 nm excitation and motorized interference filters, successfully imaging picomolar concentrations of silica-encapsulated 60-nm Au NPs amidst the broad tissue autofluorescence background in small animal^100^. This system achieved high-resolution quadruplex imaging, demonstrating its applicability for in vivo quantitative analysis.

In summary, line scanning, as observed in the SARI system, significantly enhances imaging speed and field of view while maintaining spectral resolution, which enables rapid preclinical imaging. On the other hand, the REMI system excels in ex vivo tissue imaging, allowing rapid quantification of multiple biomarkers. Widefield Raman imaging enables faster acquisition, but it sacrifices selectivity. Conversely, SERS widefield imaging provides rapid, high-resolution imaging with minimal background interference. Overall, the SARI system is recommended for fast, large-area in vivo imaging, whereas REMI and SERS widefield imaging are more suited for ex vivo and background-sensitive applications, respectively. These are summarized in Table 1.

**Table 1:** Comparison of microscope-based imaging methods: scanning versus widefield

Imaging Approach	Line Scanning (SARI System) Bohndiek et al. ^14^	Raman-Encoded Molecular Imaging (REMI) Wang et al. ^97^	Widefield Imaging Mallia et al. ^99^
Methods	A laser line is scanned along the *x* and *y* axes using galvo mirrors with a 2D EMCCD	Raster-scanned imaging of a tissue specimen using a two-axis translation stage	Background-free channel imaging using motorized narrow-bandpass filters on known SERS peaks
Excitation and signal collection	785 nm excitation (290 mW at focal plane) through a 105-μm multimode fiber Illumination and signal collection with a combination of the achromatic lenses.	785 nm excitation (10–15 mW at the tissue) Illumination and signal collection with a multimode fiber.	Excitation with a fiber-coupled diode laser at 785 nm. Signal collected by an objective and relay lens arrangement.
Field of view and imaging resolution	31 × 25 mm (>6 cm^2^) in under 30 min with a spatial resolution of 250 × 64 μm	50 × 50 mm^2^ with a laser spot diameter of 0.2–1 mm (imaging resolution) at a working distance from 2 to 6 mm	1 cm diameter uniform field of view at a working distance of 12 cm with a pixel resolution of 50 μm
Performance	Fast imaging (5 × 5 mm^2^ in 1.5 min) without needing to move the animal	An area of up to 4 × 4 cm^2^ was raster-scanned within 3 min (i.e., >5 cm^2 ^min^−1^).	Under 5 s per bandpass image (with a passband width of 4 nm, the overall filter has a transmission >95%) using a 1 cm^2^ illumination spot
Best use	Fast, large-area imaging in preclinical studies	Ex vivo biomarker analysis, e.g., intraoperative assessment of large tissue surfaces	Fast widefield imaging of thin samples. In vivo quantitative analysis with minimal background interference (autofluorescence)
Limitation	Working distance-limited to within a few millimeters of the focal plane (due to the multimode optical fiber output of the laser) Depth of penetration of the system – limited to<4 mm (while SORS: 45–50 mm)		Reduced selectivity Loss of spectral information and multiplexing ability due to spectral overlap Unsuitable for whole-body or deep tissue imaging (a practical depth of detection limit of ~5 mm in tissue, primarily due to CCD background saturation).

### Fiber-optic based configuration

In vivo detection setups require optimization for specific medical applications, such as the deep-tissue detection of SERS nanoprobe signals. Fiber-optic Raman systems, including endoscopic probes, address the limitations of microscope-based systems and are suitable for detecting and imaging tumors in internal body cavities. Fiber optics offers miniaturized and user-friendly devices for clinical applications and is usable in scanning-based and widefield imaging approaches.

Intraoperative imaging procedures for detecting tumor margins require proximity to the imaging device and no deep penetration. Conventional microscope-based systems are unsuitable, necessitating a fiber-optic spectroscopic system for real-time detection during surgery^19,94,101–105^. The Nie group reported a handheld device named SpectroPen for the intraoperative detection of malignant tumors^101^. It operates based on wavelength-resolved measurements of fluorescence and SERS signals under NIR photoexcitation, allowing precise tumor border detection.

Endoscopic imaging provides minimally invasive access to deep tissues within the body but lacks the biochemical information essential for molecular typing and early detection. Fiber-optic-based Raman spectroscopy (RS) devices have been developed for real-time multiplexed functional information during endoscopy. Zaveleta et al. designed a non-contact endoscopic imaging device to identify potentially cancerous lesions in the gastrointestinal tract using tumor-targeting SERS nanoprobes^106,107^. The device features a flexible fiber bundle for illumination and light collection, producing an illumination spot with a diameter of ~1 mm.

A rotational spectral imaging endoscope enables comprehensive imaging of SERS nanoprobes, allowing rapid circumferential scanning of luminal surfaces in hollow organs^103,108,109^. This device uses a 785 nm laser and multimode fibers for Raman signal collection, with a brushless DC motor rotating the mirror for 360-degree beam sweeping. Imaging data can be displayed as flat 2D images or projected onto a cylindrical surface for better interpretation.

SERS nanoprobe-based Raman endoscopy often struggles to identify cancerous tissues within normal-looking tissues owing to its limited field of view. Jeong et al. proposed the dual-modal imaging of SERS and fluorescence to solve this problem^19,104^. A real-time in vivo molecular imaging device named the dual-modal fluorescence Raman endoscopic system (FRES) was used with fluorescence-SERS active nanoprobes (F-SERS dots) containing tumor-specific antibodies (Fig. 4d). The fluorescent moiety can provide fast imaging ability, and SERS spectral information can compensate for multiplexing capability. Using optical fiber bundles, in vivo suspicious lesions were assessed with a non-/minimally invasive procedure in conjunction with conventional endoscopy for intraoperative real-time molecular diagnostics. A combination of fluorescence-Raman offers the specificity of RS with the versatility and speed of fluorescence imaging^110^. Additionally, it facilitates real-time fluorescence imaging to detect tumors, aids in their surgical removal and verifies clear margins afterward using RS. Using optical fiber bundles, in vivo suspicious lesions were assessed with a non-/ minimally invasive procedure in conjunction with conventional endoscopy for intraoperative real-time molecular diagnostics.

Spatially offset Raman spectroscopy (SORS) addresses the limitation of signal detection through thick tissues by collecting scattered light in regions offset from the incident light (Fig. 4e). SORS works by collecting the Raman signal from the sample at a distance from the excitation point, which results in a change in the photon path and reduces the effect of the surface layers on the Raman signal, thereby suppressing autofluorescence. By conjoining SERS substrates or nanoprobes, SORS can become a more powerful technique, called Surface-enhanced spatially offset Raman spectroscopy (SESORS)^111–116^. SESORS can detect SERS nanoprobes through 50 mm of tissue^114^, offering the potential for noninvasive cancer detection in vivo^116,117^.

Current fiber optic Raman probes are limited by the absence of imaging capabilities and rely on post-acquisition data analysis, thereby reducing their diagnostic utility. Recently, Yang et al. proposed a fiber-optic probe-based NIR Raman imaging system that integrates real-time data processing alongside augmented reality (AR) and mixed reality (MR) visualizations, potentially applicable to SERS imaging^118^. In this innovative study, Raman spectra were acquired with a handheld probe and positional data via a brightfield camera, enabling real-time reconstruction of Raman images and molecular virtual reality (VR) visualizations. A 3D surface reconstruction using photometric stereo allowed for the overlay of molecular data onto 3D surfaces, enhancing visualization. The system achieved a special resolution of 0.5 mm, surpassing the precision needed for surgical procedures, and operated at 10 Hz with excitation powers below the permissible exposure for 785 nm radiation. The photometric stereo demonstrated a depth error of less than 1 mm, confirming its clinical compatibility. Tests on 3D tissue phantoms showed minimal depth distortions (0.6 mm) and clear visualization of molecular boundaries, demonstrating successful applications in ex vivo breast cancer and lipoma biopsy samples.

In summary of “Instrumentation for In Vivo SERS Application”, advancements in Raman instrumentation and imaging have significantly improved in vivo SERS applications. Optimizing components such as excitation sources, detectors, and imaging systems enhances the sensitivity, specificity, and spatial resolution. Developments in both microscope-based and fiber-optic configurations have broadened SERS imaging capabilities, offering detailed molecular insights in preclinical and clinical settings. Despite these advances, future efforts should prioritize refining these systems to lower economic barriers, enabling their use in various clinical and research settings while ensuring accessibility for users with diverse abilities. Enhancing user-friendliness involves developing intuitive interfaces, streamlining workflows, and improving guidance for medical professionals, ensuring that the systems remain reliable and easy to operate, even for those without specialized expertise in spectroscopy.

## In vivo applications of SERS-based techniques

This section explores various SERS-based applications for in vivo detection, diagnosis, and imaging for intraoperative guidance. Here, we discuss the basic concepts of detection and diagnosis using in vivo SERS to describe the compatible capability of in vivo SERS to other techniques. We also describe the main strengths of in vivo SERS imaging, multiplex capability, and intraoperative guidance.

### In vivo sensing and diagnosis

**Fig. 5:**
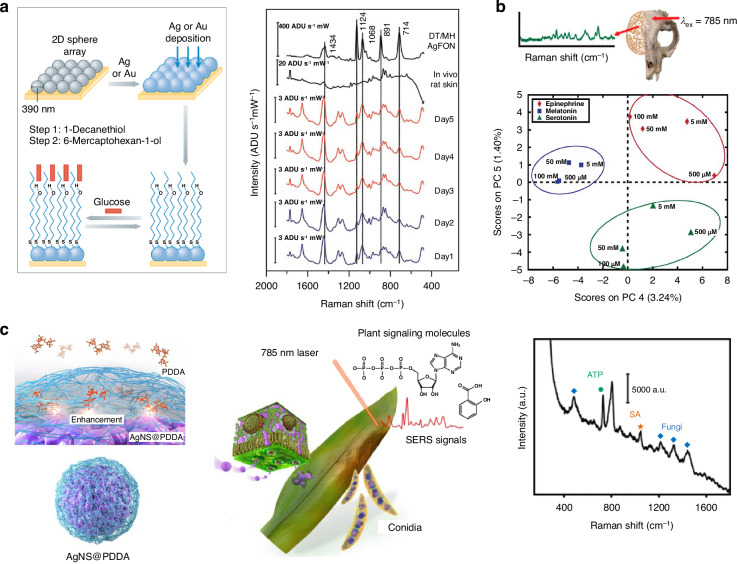
SERS-based in vivo detection and diagnosis. **a** In vivo glucose monitoring based on SESORS detection using silver (or gold) film over nanosphere substrate implanted in a live rat. Reproduced from refs. ^120^ and ^121^ with permission from Springer Nature and Copyright 2011 American Chemical Society, respectively. **b** SERS measurements of neurotransmitters (melatonin, serotonin, and epinephrine) in a brain tissue mimic through a cat skull. Reproduced from ref. ^124^ with permission from Copyright 2017 American Chemical Society. **c** Schematic illustration of early diagnosis of fungal disease in living crop plants with the Raman enhancement on the surface of AgNS@PDDA. Raman spectra show the SERS signals of extracellular adenosine triphosphate (ATP), salicylic acid (SA), fungi. Reproduced from ref. ^128^ with permission from Springer Nature

SERS-based in vivo detection offers noninvasive approaches for clinical diagnostics across a broad spectrum, from human diseases to plant health. One of the most significant applications in clinical diagnostics is glucose sensing^119^, driven by the need for non-invasive methods to measure glucose levels as an alternative to daily finger prick tests required by conventional blood glucose meters. Noninvasive in vivo monitoring of blood glucose levels using SESORS was explored, even in the early stages of SERS application (Fig. 5a)^111,120,121^. In a specific investigation, a silver film over nanosphere SERS substrate was fabricated and functionalized with a mixed self-assembled monolayer. This substrate was subsequently implanted subcutaneously in a rat. Employing SESORS, SERS spectra of glucose in the interstitial fluid were acquired from the substrate through the skin. Yang et al. reported glucose sensing in the aqueous humor of fresh ex vivo rabbit eyes using a Raman-mode-constraining approach^122^. Polydimethylsiloxane was used to fabricate the implant mounts for the SERS disks used in the study. Fast and continuous glucose sensing within a physiologically relevant range (0.1–30 mM) was demonstrated by tracking glucose-induced shifts in the SERS spectra of mercaptophenylboronic acid.

Noninvasive detection of neurotransmitters through the skull could profoundly enhance our comprehension of brain function and neurological disease research^123^. Precise understanding of neurochemical properties and local concentrations is crucial, demanding a noninvasive method for measuring these substances within the brain. Sharma et al. utilizing SESORS, successfully detected neurotransmitters in a brain tissue phantom through a cat skull at concentrations as low as 100 µM (Fig. 5b)^124^. More recently, Vo-Dinh et al. employed an inverse SORS approach to recover SERS spectra using a monkey skull^125^. In their experiment, Au nanostars were suspended in an agarose gel phantom and shielded by a 5 mm thick monkey skull. Additionally, SESORS has been used to detect ex vivo multicellular tumor spheroids in 15 mm of tissue using a handheld SORS spectrometer, allowing successful detection of 3D breast tumor models^116^.

Detecting the active forms of endogenous signaling molecules in living plants is challenging because of their low concentrations and the presence of many other organic compounds^126,127^. Although SERS is a powerful analytical tool, its application to detecting plant signaling molecules in living plants remains unexplored. However, in a recent study, Son et al. introduced a nondestructive SERS nanosensor for real-time detection of multiple stress-related endogenous signaling molecules in living plants (Fig. 5c)^128^. Ag nanoshells with a highly bumpy surface containing hot spots were modified using a water-soluble cationic polymer, poly(diallyldimethylammonium chloride) (PDDA), to increase water compatibility and bring signaling molecules close to the nanosensor surface. This in vivo SERS nanosensor, effectively suppressing chlorophyll autofluorescence with 785 nm excitation, was directly injected into living plants to monitor signaling molecules such as adenosine 5’-triphosphate (ATP), salicylic acid (SA), indole-3-acetic acid (IAA), and folic acid (FA). The authors successfully monitored stress-related molecules in *Nasturtium officinale*, *Triticum aestivum L*., and *Hordeum vulgare L*. under abiotic and biotic stresses, indicating the possible onset of plant diseases.

### Biomolecule screening and multiplex imaging

**Fig. 6:**
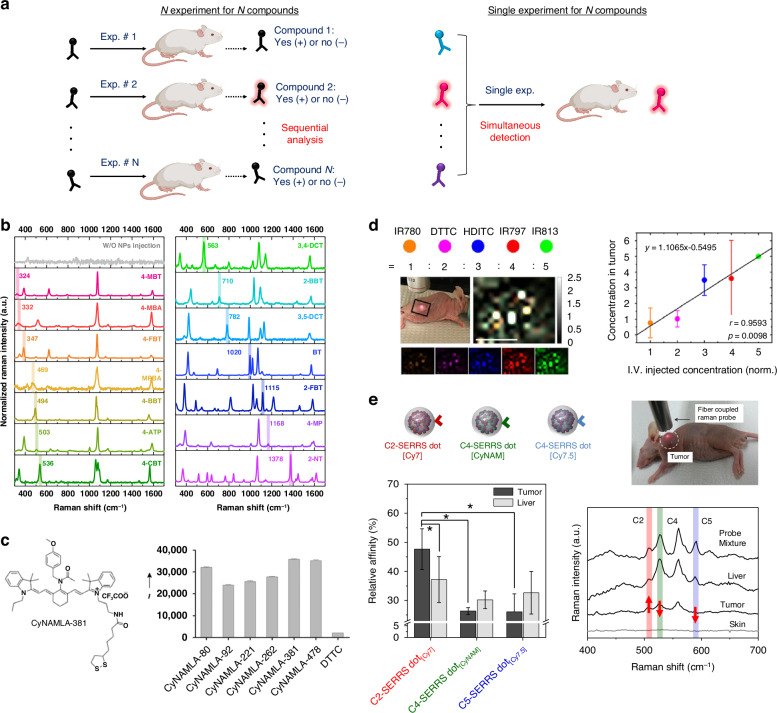
In vivo multiplexed detection and quantification. **a** Comparison of multiplex analysis with sequential analysis. Multiplex detection can significantly reduce the number of experiments. **b** Combinatorial synthesis and screening of Raman label compounds with multiplex capability and high sensitivity. Adapted from ref. ^22^ under (CC BY 4.0). **c** In vivo five-plex ratiometric SERS imaging of tumor bearing mouse injected with different concentrations of SERS probes. Reproduced from ref. ^59^ with permission from John Wiley and Sons. **d** Unique SERS spectra of 14 different Raman label compounds at 785-nm photoexcitation. Each compound showed distinct bands. Reproduced from ref. ^90^ with permission from Copyright 2021 American Chemical Society. **e** In vivo multiplexed antibody validation strategy based on ratiometric quantitative SERS analysis. Reproduced from ref. ^6^ with permission from John Wiley and Sons

The preparation and screening of molecular libraries with high multiplexing capabilities have advanced rapidly for drug discovery, disease biomarker identification, gene screening, and biomolecular profiling. Selecting lead compounds from a library is crucial but labor-intensive, especially when each compound is verified individually (Fig. 6a, left). Several encoding/decoding methods, including chemical, graphical, and optical techniques, have been developed to simplify and accelerate compound identification in large libraries^129–131^.

SERS tagging, also known as colloidal barcoding, is a powerful tool for identifying lead compounds in high-throughput screening bioassays because of its narrow spectral width. Similarly, SERS nanoprobes offer effective and fast screening and multiplexed biomolecular detection in vivo (Fig. 6a, right). Many commercially available aromatic compounds with different functional groups, such as thio, disulfide, amino, cyano, and isothiocyanato moieties, are considered suitable RLCs (Fig. 6b)^11,64,66,129,132^. Furthermore, combining RLCs during SERS tag fabrication has yielded sufficient unique codes^63,130,133^. Theoretically, two RLC combinations with only 30 RLCs can generate more than 10 trillion codes. However, when multiple SERS tags are located within the micron-sized measurement spots, the concurrent labeling of RLCs in single NPs can be indistinguishable from others nearby^64,130,133^.

To expand the multiplexity of SERS probes with a limited number of RLCs^11,23^, the Chang group synthesized an RLC library based on a heptamethine indocyanine dye structure via combinatorial solid-phase synthesis. Among the 80 derivatives, several compounds, such as CyNAMLA, demonstrated high SERS activity, offering 12-fold higher sensitivity than commercially available DTTC (Fig. 6c)^59^. This method was extended to synthesize CyNAMLA derivatives for multiplex detection of cancer markers^60^. Harmsen et al. rationally designed and synthesized chalcogenopyrylium-based dyes as NIR-absorbing RLCs with high affinity for the Au NP surface to achieve the SERRS effect^134,135^. The SERRS nanoprobe, labeled with pyrylium dye, provides a limit of detection as low as the attomolar range, allowing for ultrasensitive detection of multiple tumor and disease markers in vivo.

The Gambhir group reported pioneering studies, including the noninvasive multiplex detection of cancer markers^11,107^. For example, five multiplexing ratiometric and longitudinal SERS images were obtained in subcutaneously implanted tumors in nude mice (Fig. 6d)^90^. A total of 600 fmol of the five-color SERS nanoprobes were injected simultaneously into tumor-bearing mice, and SERS signals were observed in the tumor area under 785 nm NIR laser excitation. This approach facilitates the noninvasive assessment of multiple biological targets within the tumor microenvironment in living subjects. In another practical application, Kang et al. demonstrated a NIR-SERRS dot-based ratiometric quantification method for validating anti-tetraspanin-8 (TSPAN8) antibodies in human colon cancer in vivo (Fig. 6e)^6^. Three TSPAN8 antibody candidates with varying affinities were conjugated to the corresponding nanoprobe, NIR-SERRS dot, respectively. A mixture of TSPAN8 antibody-conjugated NIR-SERRS dots was applied to HCT8 cells in vitro and intravenously administered to human colon cancer-bearing mice. This single-mouse validation allows cost-effective and accurate multiplexing measurements, which are essential for antibody-based drug development. This study highlighted the potential of SERS-based techniques for validating and characterizing antibody candidates, thereby facilitating their translation into clinical applications in cancer diagnosis and therapy.

### Intraoperative guidance

Surgical resection stands as the cornerstone of cancer treatment, playing a crucial role in enhancing prognosis and survival outcomes by ensuring complete tumor removal. Yet, the challenge lies in detecting microscopic tumor residues amidst normal tissues, which often eludes surgeons and may lead to recurrence. This underscores the critical need for intraoperative imaging techniques that enable precise assessment of tumor margins and effective removal of tumor tissues during surgery. Various intraoperative imaging methods have been proposed, including photoacoustic and fluorescence imaging. These techniques harness tissues’ inherent optical properties or external contrast agents. However, they frequently encounter limitations such as reduced sensitivity and specificity. Factors contributing to these challenges include tissue autofluorescence, limited resolution, and shallow penetration depth, complicating the accurate delineation of tumor margins.

**Fig. 7:**
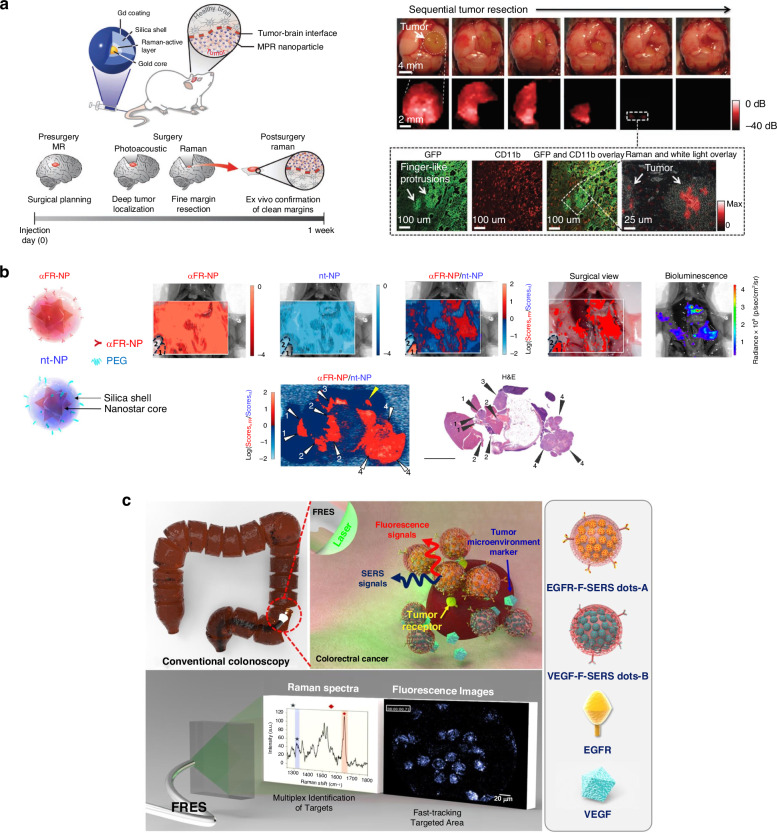
SERS-guided tumor margin determination in intraoperative scenario. **a** Demonstration for sequential tumor resection and margin determination of brain tumor with SERS-guidance until the entire tumor had been removed. Reproduced from ref. ^13^ with permission from Springer Nature. **b** A ratiometric SERS imaging for determination of disseminated ovarian cancer lesions. SERS ratiometric imaging with targeted and non-targeted SERS nanoprobes can visualize the only tumor regions and eliminate signal of nonspecific distribution. Reproduced from ref. ^77^ with permission from Copyright 2017 American Chemical Society. **c** fluorescence-Raman simultaneous detection strategy on colon cancer model to facilitate molecular characterization of a tumor. Reproduced from ref. ^104^ under (CC BY 4.0)

To overcome these limitations, in vivo SERS imaging has emerged as a promising tool for real-time, noninvasive microscopic detection during surgical procedures^136^. SERS nanoprobes with high sensitivity and specificity can accurately identify tumor boundaries. Kircher et al. reported unique trimodal NPs (MRI-photoacoustic-Raman, MPR NPs) that precisely delineated brain tumor margins both preoperatively and intraoperatively in mice (Fig. 7a)^13^. The MPR NPs consist of a 60-nm gold core surrounded by RLCs and a 30-nm silica shell. When administered to a mouse bearing a brain tumor, these NPs circulate in the bloodstream, traverse the disrupted blood-brain barrier, and accumulate within the tumor tissue. During surgery, photoacoustic imaging (PAI) guides bulk tumor resection, while Raman imaging aids in removing remaining microscopic tumors. Each resected brain tumor section is subject to histological analysis, revealing infiltrative tumor patterns with detectable Raman signals indicative of MPR NP presence. MPR NPs enable comprehensive localization of whole-brain tumors, facilitate high-spatial-resolution 3D imaging, and offer high sensitivity and specificity for surface imaging of tumor margins. Furthermore, a handheld Raman scanner has been documented to aid in detecting additional microscopic foci of glioblastoma^102,137^, providing near real-time detection of microscopic tumor extents that are not visible on a static micro-Raman device.

The Ye group demonstrated rapid and accurate intraoperative positioning of sentinel lymph nodes (SLNs) for minimally invasive surgery using a handheld Raman scanner^138^. The developed SERS GERTs were used to amplify SERS signals through hotspots. This SERS-based SLN identification method outperformed conventional methods such as methylene blue staining and fluorescence tracking with indocyanine green, providing better guidance for surgeons. Furthermore, Jeong et al. integrated simultaneous fluorescence-SERS imaging into colonoscopy (standard white-light endoscopy) to facilitate in vivo molecular diagnosis and excision of suspected colorectal cancer lesions^19,104^. Fluorescent-SERS nanoprobes (F-SERS dots) were employed for real-time fast-tracking of targeted areas through fluorescence scanning and identifying multiple targets via RS^139^. The two antibody-conjugated fluorescent-SERS dots (F-SERS dots) targeted EGFR tumor markers within tumor cells and tumor-secreted VEGF in the tumor microenvironment. This fluorescent-SERS endoscopic technique allows for simultaneous capture of multiple molecular characteristics of a tumor during colonoscopy. Applying a topical spray of F-SERS dots directly onto small tumors can minimize potential toxicity in clinical settings.

To enhance specificity and reduce nonspecific off-target binding during intraoperative identification of tumorous lesions, a ratiometric approach with multiplexed SERS nanoprobes has been implemented^77,97,140^. Oseledchyk et al. demonstrated a ratiometric imaging strategy for accurate identification of peritoneal dissemination in ovarian cancers using Au nanostars labeled with IR780 (αFR-NP in Fig. 7b) or IR140 (nt-NP in Fig. 7b)^77^. These SERRS nanoprobes were functionalized with a folate receptor-targeting antibody for targeted NPs (αFR-NP) or with PEG for non-targeted NPs (nt-NPs). Following intraperitoneal administration of a mixture of these SERRS-NPs to mice bearing folate receptor-overexpressing ovarian cancer, luciferin injection was performed, and the abdominal cavity was scanned using a Raman microscope to assess the ratiometric signal (Score αFR-NP/Score nt-NPs) against a bioluminescence imaging map (Fig. 7b). Despite varied probe distribution in the peritoneal cavity, the ratiometric algorithm accurately identified tumorous lesions. Histological analysis confirmed that nearly all identified lesions were indeed tumors, validating the high accuracy of this approach. Similarly, Bao et al. employed a ratiometric strategy using folic acid-functionalized targeted or non-targeted SERS nanoprobes with nanogaps (FA-GERTs and Nt-GERTs) for intraoperative detection of metastatic lymph nodes^141^.

### Multifunctional SERS nanoprobes for cancer therapy

**Fig. 8:**
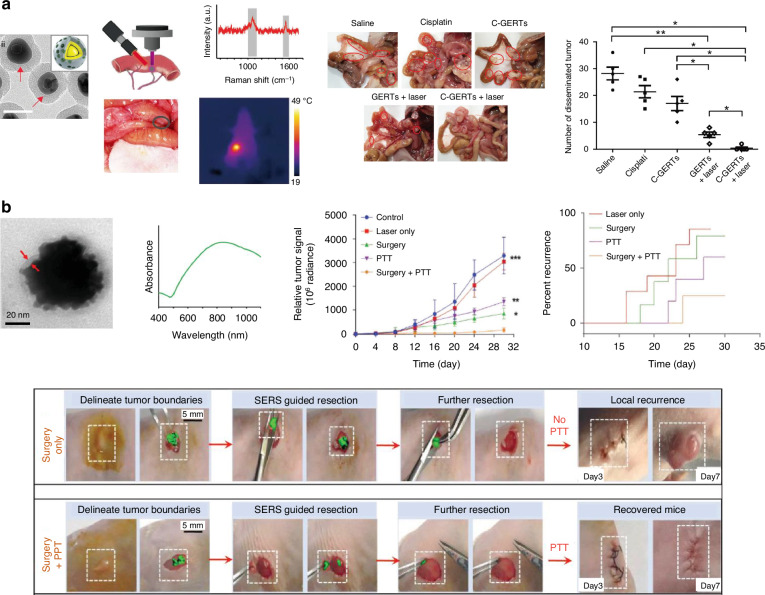
SERS-guided cancer therapy with multifunctional nanoprobes. **a** Intraoperative SERS-guided chemo-photothermal therapy of disseminated ovarian cancer. Cisplatin releasing and heat generation upon 808 nm laser irradiation (3 W cm^−2^, 5 min) Reproduced from ref. ^144^ with permission from John Wiley and Sons. **b** Combination of SERS-guided tumor resection and post-surgical adjuvant photothermal therapy to prevent recurrence and prolong the survival rate. Reproduced from ref. ^145^ with permission from John Wiley and Sons

Most SERS nanoprobes, typically composed of metals such as Au and Ag, exhibit high photothermal conversion efficacy. This property has enabled the development of ultrasensitive SERS nanoprobes that integrate diagnostic detection with PTT and chemotherapy, thus facilitating the detection and elimination of disseminated microtumors^110,142,143^. Zhang et al. developed multifunctional SERS nanoprobes for Raman-guided intraoperative imaging and synergistic therapy for ovarian cancer (Fig. 8a)^144^. They synthesized cisplatin-loaded GERTs (C-GERTs) for the detection and chemo-PTT of abdominal microtumors. C-GERTs consist of core-shell Au NPs with embedded RLCs and a mesoporous silica layer loaded with cisplatin exhibit high specificity and sensitivity in identifying microtumors as small as 0.1 cm in diameter using SERS signals under 808 nm laser irradiation. Moreover, a significant therapeutic effect was observed in most mice treated with C-GERTs and laser irradiation, highlighting their potential for real-time intraoperative detection and treatment of unresectable tumors. This approach enables Raman-guided localized PTT and chemotherapy, allowing precise targeting of cancer cells while sparing normal tissues.

In 2021, Wei et al. reported multifunctional urchin-like Au NPs conjugated with an NIR dye as RLCs for SERS-guided tumor surgery and tumor-selective PTT^145^. They demonstrated successful removal of subcutaneous and orthotopic ovarian and colon tumors under SERS guidance, ensuring no detectable SERS signals remained in the tumor bed post-surgery. Following surgery, they utilized 808 nm laser-induced PTT to selectively ablate tumor margins. These findings indicate that combining SERS-guided surgery with post-surgical PTT can potentially reduce tumor recurrence and enhance survival rates.

Recently, SERS-guided PTT in the NIR-II window has shown promise owing to its satisfactory photothermal conversion efficiency and improved penetration depth with a 1064 nm laser^146^. Compared to 808 nm laser, 1064 nm laser irradiation offers deeper tissue penetration (up to 10 mm), higher photothermal conversion efficiency (~67.1%), and better therapeutic efficacy for solid colorectal neoplasms. The benefits of SERS and PTT in the NIR-II window are discussed in the following sections.

## Challenges for clinical implementation

Although SERS has been widely recognized as an ultrasensitive imaging tool, visible-light-responsive SERS nanoprobes face significant limitations in vivo because of the heavy absorption and scattering of visible light by biological components such as hemoglobin, water, melanin, and fat, and extensive background autofluorescence from tissue chromophores^27,28^. Consequently, the NIR-I window (650–900 nm) is preferred for in vivo SERS imaging and therapy because of its accessibility and cost-effectiveness, although it still has limitations in terms of penetration depth compared with the NIR-II window (1000–1700 nm).

**Fig. 9:**
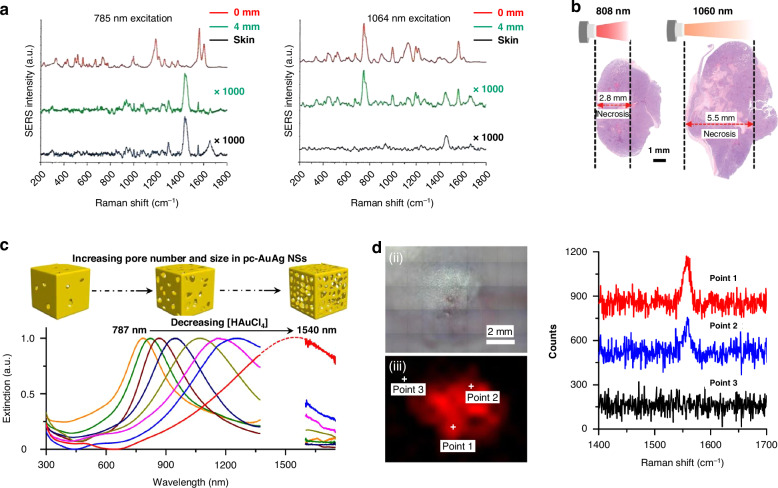
In vivo SERS detection in NIR-II range. **a** comparison of tissue penetration capability of SERS at 785 and 1064 nm photoexcitation. Reproduced from ref. ^28^ with permission from Elsevier. **b** Comparison of effective penetration depth between 808 nm and 1064 nm wavelengths in photothermal cancer therapy. Reproduced from ref. ^146^ with permission from John Wiley and Sons. **c** Tuning plasmon resonance wavelength of Au-Ag nanoframe from 700 to 1400 nm in NIR-I and NIR-II range and **d** its sensitive detection of microtumors in living animals up to 100 cells/mL in NIR-II. Reproduced from ref. ^147^ under (CC BY 4.0)

Recent advancements in SERS imaging using the NIR-II window have significantly improved tissue penetration and resolution. This progress is attributed to reduced light scattering, minimal light absorption, and extremely low autofluorescence levels (Fig. 9a, b). For instance, porous cubic Au/Ag alloy nanoshells exhibit exceptional NIR-II plasmonic properties, which can be finely tuned across a broad spectral range by adjusting their pore characteristics (Fig. 9c)^147,148^. Utilizing these NIR-II-responsive Au/Ag alloy nanoshells, unique Raman signals were detected exclusively within tumor tissue, with no signals observed in adjacent healthy skin, allowing for clear delineation of the tumor border (Fig. 9d). When SERS nanoprobes aggregate, they may reduce recovery rates, potentially causing toxicity. To enhance their recovery in various biological samples, several strategies have been developed^149^. These include polymer coating and charge variations of SERS nanoprobes, which can improve their reliability and efficacy in practical applications.

Other considerations include tumor targetability and toxicity. The ability of the SERS nanoprobes to enter cancerous lesions is determined by their physicochemical properties and biological barriers. Extending blood circulation is crucial for increasing the likelihood of reaching tumor tissues^150,151^. SERS nanoprobes from non-aggregated Au NPs smaller than 20–30 nm can lead to low sensitivity owing to their low SERS cross-sections^152–154^. However, NPs larger than 20 nm may be rapidly eliminated via Kupffer cell uptake^155^. Researchers have developed several strategies to enhance safety for clinical applications, such as surface coating, altering charge density/hydrophilicity/hydrophobicity, and introducing naturally derived cell membranes^156^. These approaches aim to mitigate potential toxicity and adverse effects and significantly improve biodistribution and pharmacokinetic properties. These physicochemical properties are essential for minimizing potential toxicity and achieving precise tumor targeting, ultimately facilitating the clinical translation of SERS nanoprobes.

Ensuring the reproducibility and robustness of SERS technology in clinical settings needs the establishment of rigorously defined protocols and calibration methods. These standards must be collaboratively formulated with contributions from both academic and industry experts to adapt to different laboratory environments and regulatory requirements. By focusing on stringent quality control measures and fostering an environment of continuous improvement and validation, the field can move towards standardized SERS applications that offer reliability and confidence in clinical diagnostics.

The integration of advanced computational techniques, such as artificial intelligence (AI) and machine learning (ML), with SERS technology holds the potential to revolutionize in vivo imaging. These technologies could enable the analysis of complex datasets generated by in vivo SERS imaging, enhancing the ability to distinguish between healthy and diseased tissues with high precision. The application of ML algorithms could make the interpretation of SERS data more efficient, potentially providing clinicians with tools that would improve diagnostic accuracy and patient outcomes. Moving forward, the challenge will be to develop and refine these computational models to fully use deep learning capabilities in real-time clinical scenarios.

Exploring the integration of SERS with advanced imaging modalities such as optical coherence tomography and PAI is opening new avenues for potential multimodal imaging approaches that could combine structural, functional, and molecular insights. This constructive collaboration has the potential to offer a more holistic view of disease mechanisms at both the macro and micro levels, which could significantly enhance diagnostic capabilities in the future. Developing multimodal platforms that incorporate SERS is expected to require innovative engineering solutions to ensure seamless operation and effective data integration, thereby maximizing the clinical utility of these technologies. Ongoing research in this area is crucial to realize these possibilities and transform them into practical clinical applications.

Lastly, to further bridge the gap between the promising capabilities of in vivo SERS and its clinical translation, comprehensive studies are essential. These studies should meticulously address the pharmacokinetics, biodistribution, and biocompatibility of SERS nanoprobes, particularly in the context of the NIR-II window. By conducting detailed investigations into these areas, researchers can overcome the fundamental challenges posed by biological components and tissue interactions, thus paving the way for practical clinical applications. Such efforts will ensure that SERS nanoprobes not only prove superior imaging capabilities but are also safe and effective for human use.

## Conclusions

This review comprehensively discusses the advancements and applications of in vivo SERS techniques, highlighting their significant potential across various biomedical fields. For in vivo applications, spontaneous RS offers excellent chemical specificity but is limited by weak signals, slow imaging, and shallow penetration depths. However, advances in spontaneous RS, including optimized excitation sources and enhanced detection techniques, have improved its utility in both in vivo and ex vivo biomedical imaging, particularly for surface-level tissue analysis. Despite these improvements, SERS techniques have made more significant strides in overcoming the inherent limitations of spontaneous RS in biomedical imaging. SERS nanoprobes have been developed to enhance sensitivity, reproducibility, multiplexing capacity, multifunctionality, and biocompatibility.

Advances in NIR excitation and optically tuned SERS substrates have improved tissue penetration and reduced background interference. Instrumental innovations, including microscope-based configurations for scanning and widefield imaging, as well as flexible and precise fiber-optic-based setups, have established in vivo SERS imaging as a powerful tool in biomedicine. SERS-based detection has demonstrated potential in cancer detection, intraoperative guidance, and real-time monitoring of biological processes. Multifunctional SERS nanoprobes have enabled precise tumor imaging and targeted therapies, enhancing the efficacy of photothermal and chemotherapeutic approaches.

Despite substantial advancements, several challenges persist, such as enhancing penetration depth, reducing acquisition times for high-quality images, minimizing background autofluorescence, and ensuring biocompatibility, which are crucial for the clinical application. The future of in vivo SERS imaging in the NIR-II window holds promising prospects, supported by ongoing research aimed at refining nanoprobe design, optimizing imaging methodologies, and developing cost-effective real-time instrumentation. Machine learning and AI are expected to further enhance data interpretation, making diagnosis faster and more accurate. Continued innovation will further establish SERS imaging as an indispensable tool for personalized medicine and targeted cancer therapy.
